# Expression of Pfkfb isoenzymes during in vitro differentiation of mouse embryonic stem cells into insulin-producing cells

**DOI:** 10.55730/1300-0144.5725

**Published:** 2023-08-11

**Authors:** Saime GÜZEL, Abdullah YALÇIN, Yunus GÜRPINAR, Sabire GÜLER

**Affiliations:** 1 Department of Biochemistry, Faculty of Veterinary Medicine, Bursa Uludağ University, Bursa, Turkiye; 2 Research Center for Translational Medicine, Koç University, İstanbul, Turkiye; 3 Department of Histology & Embryology, Faculty of Veterinary Medicine, Bursa Uludağ University, Bursa, Turkiye

**Keywords:** Mouse embryonic stem cells, insulin-producing cells, differentiation, 6-phosphofructo-2-kinase/fructose-2,6-bisphosphatase

## Abstract

**Background/aim:**

Type 1 diabetes mellitus (T1DM) is caused by the autoimmune-mediated destruction of insulin-producing cells (IPCs) and still has no effective cure. Better understanding of the molecular mechanisms involved in the differentiation of embryonic stem cells (ESCs) into IPCs may help us improve the therapeutic strategies for treating T1DM. 6-phosphofructo-2-kinase/fructose-2,6-bisphosphatases (Pfkfb1–4) are key regulators of glucose metabolism. Although Pfkfb3 has been shown to be required for the growth of early differentiated mouse ESCs (mESCs), more studies are needed to further assess the roles of Pfkfb isoenzymes in embryonic development and differentiation, particularly into specific cell types. In this study, we aimed to elucidate the changes in the expression of Pfkfb isoenzymes on the differentiation of mESCs into IPCs.

**Materials and methods:**

A 3-step protocol was used to differentiate R1 and J1 mESCs into IPCs. The changes in the gene expression of MafA, MafB, Ins2, and Nkx6.1 (IPC specific markers) and Pfkfb1–4 were analyzed using real-time quantitative polymerase chain reaction (qPCR). Insulin expression and secretion were determined by immunofluorescence (IF) staining and the enzyme linked immunosorbent assay (ELISA), respectively.

**Results:**

Upon differentiation, the IPC specific markers in differentiated cells were upregulated. Continued differentiation was confirmed by the development of insulin-positive islet-like clusters that secreted insulin in response to glucose uptake. Expressions of the Pfkfb2 and Pfkfb3 isoenzymes were markedly increased in various stages of differentiation.

**Conclusion:**

These findings suggest that Pfkfb2 and Pfkfb3 may impact the differentiation of mESCs into IPCs and the regulation of the insulin response to glucose levels. This study also lays a foundation for researchers to further probe the roles of Pfkfb isoenzymes on the differentiation of mESCs into IPCs and may open new avenues for regenerative medicine.

## 1. Introduction

Diabetes mellitus (DM) is a chronic disease characterized by a disorder in carbohydrate, fat and protein metabolism that occurs as a result of hyperglycemia due to insufficient insulin hormone. Type 1 DM (T1DM), the most severe form, is an autoimmune chronic process that is associated with insulin deficiency or absence as a result of the destruction of insulin-producing cells (IPCs) in the pancreatic islets. Even though exogenous insulin is used to treat T1DM, patients can become insulin-dependent, leading to attacks of hypoglycemia or hyperglycemia and poor quality of life [1]. Although pancreas and islet cell transplantations have been attempted, the problems of donor shortages, requirements for major surgery, tissue rejection, and the need for life-long immunosuppressive drugs limit the therapeutic use of both options [2,3]. In recent years, a promising treatment approach is the use of pluripotent embryonic stem cells (ESCs) to generate an unlimited source of IPCs [2,4,5]. However, stem cell-based therapies have been rather inefficient due to the inability of the cells to produce insulin at the expected level and time. Therefore, detailed studies are needed on the possible roles of metabolic genes in the development of pancreatic islet cells, which function as glucose sensors.

Cells use metabolic pathways to maintain their vital functions and to obtain the energy required at varying levels in different periods of embryonic development. For example, mammalian cells produce ATP by modulating the ratios of glycolysis and oxidative phosphorylation (OXPHOS). While mouse embryonic stem cells (mESCs) and human embryonic stem cells (hESCs) generate energy mostly by the conversion of glucose to lactate enzymatically, somatic cells rely on OXPHOS as their primary source of energy production [6]. However, unlike many terminally differentiated cell types, IPCs generate a significant portion of their required energy via glycolysis. Furthermore, the intermediates of anaerobic glycolysis have been shown to be required for β cell glucose sensing [7]. In addition, the inhibition of liver kinase B1 (LKB1), which is the activator of the glycolytic stimulator adenosine monophosphate-activated protein kinase (AMPK), causes an increase in glucose-stimulated insulin secretion in β cells [8].

The 6-phosphofructo-2-kinase/fructose-2,6-bisphosphatase (Pfkfb) family of enzymes (Pfkfb1, Pfkfb2, Pfkfb3, and Pfkfb4) are responsible for the synthesis and degradation of fructose 2,6-bisphosphate (F2,6BP) via their kinase and phosphatase domains, respectively [9]. F2,6BP is the most potent allosteric activator of 6-phosphofructo-1-kinase (PFK-1), which catalyzes the irreversible conversion of fructose-6-phosphate (F6P) and ATP into fructose-1,6-bisphosphate (F1,6BP), a key control point in glycolysis [10]. Besides the role of Pfkfb isoenzymes in the glycolytic activity, there are also studies regarding their functions in mouse embryonic development [11], tumor cell proliferation [12], cell survival [13], and cell differentiation [14]. Our recent study has demonstrated that Pfkfb3 may be required for the differentiation and growth of mESCs [15]. In addition, it has been reported that Pfkfb3 is required for the proliferation and survival of tumor cells, which are similar to stem cells in many ways [12,16,17]. Arden et al. [18] also reported that Pfkfb2 and Pfkfb3 isoenzymes, which are phosphorylated by AMPK to activate their kinase function (F2,6BP production), are required for glucose-dependent insulin secretion from pancreatic β cells. However, this study was performed in terminally differentiated cells, and no study has been done on the potential function and changes in expressions of Pfkfb isoenzymes during the differentiation of mESCs into IPCs.

The current study was carried out to determine the changes in expression of the Pfkfb family of enzymes at different stages of in vitro differentiation of mESCs into IPCs so as to contribute to the elucidation of the molecular mechanisms in this process.

## 2. Materials and methods

### 2.1. ES cell culture and chemicals

Pluripotent J1 (ATCC SCRC-1010) and R1 (ATCC SCRC-1011) mESCs were used as models in this study. The mESCs were cultured using sterile cell culture flasks (Corning, Netherlands) coated with 0.1% gelatin (Sigma, Germany), at 37 °C in a humidified atmosphere containing 5% carbon dioxide (CO_2_). Dulbecco’s modified Eagle’s medium (DMEM) (Sigma, Germany) supplemented with 15% ESC-qualified fetal bovine serum (Sigma, Germany), 0.1 mM MEM nonessential amino acids (Sigma, Germany), 0.1 mM 2-mercaptoethanol (Sigma, Germany), L glutamin (Sigma, Germany), 100 U/mL penicillin-100 microgram/mL (ug/mL) streptomycin mixture (Sigma, Germany), and 1000 units/mL of recombinant mouse leukemia inhibitory factor (LIF) was used for growing pluripotent mESCs. Cells were passaged every third or fourth day using trypsin EDTA, and the media were changed every other day.

### 2.2. Immunofluorescence (IF) analysis

An IF assay was performed to examine the LIF activity. The cells on culture chamber slides (BD Biosciences, USA) were fixed in 4% paraformaldehyde (Merck Millipore, USA) and IF staining was performed according to the manufacturer’s protocol (Cell Signaling, USA). The cells were incubated overnight at 4 °C with primary antibodies of the pluripotency markers Oct-4 (Cell Signaling 2840, USA) and Sox-2 (Cell Signaling 4900, USA) and probed with Alexa-Fluor 488-conjugated secondary antibody for 1 h at room temperature (Cell Signaling 4408, USA).

In addition, an IF assay was applied using Alexa Fluor-labeled insulin monoclonal antibody (Insulin Ab, Alexa Fluor 488, Thermo Fisher Sci., 53-9769-80, USA) to confirm insulin expression in the differentiated cells. All fluorescent images were visualized using an EVOS imaging system (Thermo Fisher Sci., USA).

### 2.3. In vitro differentiation of mESCs into IPCs

In vitro differentiation of the mESCs into IPCs was performed based on the protocol of Schroeder et al. [19] with minor modifications. Briefly, this protocol consists of 3 steps: (i) the formation of embryoid bodies (EBs), (ii) the spontaneous differentiation of EBs into progenitor cells of ecto-, meso-, and endodermal lineages, and (iii) the stimulation of differentiation of the early progenitors into the pancreatic precursor cells. In the first step of differentiation, the hanging drop method was used for EB formation. For this purpose, cells were suspended in a LIF-free medium and inoculated as 27 μL (900 cells) drops onto the lids of bacteriological dishes containing 10 mL PBS, and the lids were turned over to cover the plates. Approximately 50–55 drops were placed in a 100 mm dish. Thus, this technique generates one EB per drop and a more homogeneous fraction of EBs. At the end of the 3-day incubation period, the EBs were transferred to the ultralow attachment 96-well plates as 1 EB in each well and incubated again for 3 days (total 6 days). The EBs were transferred onto the gelatin-coated culture plates and were cultured for 9 more days while changing the medium every other day (6 + 9 days). After this period, the cells were removed for specific differentiation and transferred into Matrigel-coated 60-mm cell culture dishes. To induce specific differentiation, the cells were incubated in a medium including DMEM/F12, 20 nanomolar (nM) progesterone (Sigma, Germany), 100 μM putrescine (Sigma, Germany), 1 μg/mL laminin, 10 mM nicotinamide (Sigma, Germany), 25 μg/mL insulin (Sigma, Germany), 30 nM Na_2_SeO_3_ (Sigma, Germany), 50 μg/mL transferrin (Sigma, Germany), B27 medium (Invitrogen, Carlsbad, CA, USA) and pen/strep at 37 °C and 5% CO_2_ (6 + 28 days). All microscopic images were captured at 6, 6 + 9, 6 + 16, and 6 + 28 days. The medium was changed every second or third day. Pluripotent mESCs were grown in the presence of LIF as the control group during the differentiation stages.

### 2.4. Real-time quantitative polymerase chain reaction (qPCR)

The differentiated cells (6 + 9, 6 + 16, and 6 + 28 days) and the pluripotent mESCs were lifted with trypsin for total RNA isolation, centrifuged at 300 × g and washed twice with cold PBS.

Total RNA isolation and complementary DNA (cDNA) synthesis were performed using commercial kits (Thermo Fisher Sci. and Applied Biosystems, USA) following the manufacturer’s directions. qPCR analyses were carried out using StepOne Plus (Thermo Fisher Sci., USA) with gene expression master mix (Applied Biosystems, USA) and gene-specific TaqMan probes (Thermo Fischer Sci., USA, Cat. nos: Pfkfb1, Mm01256237_m1; Pfkfb2, Mm00435575_m1; Pfkfb3, Mm00504650_m1; Pfkfb4, Mm00557176_m1; Nkx6.1, Mm00454961_m1; MafA, Mm00845206_s1; MafB, Mm00627481_s1; Ins2, Mm00731595_gH; Tubb5, Mm00495806_g1). Tubb5 was used as a housekeeping gene control for normalization of the cDNA. Cycle threshold (CT) values were taken from the qPCR reactions and up/down regulation of the genes of interest was determined using the ΔΔCT method with undifferentiated mESCs as a baseline [20].

### 2.5. Glucose-stimulated insulin secretion

Differentiated cells at 6 + 28 days and mESCs grown in the presence of LIF as a control were cultured in insulin-free medium for 36 h. Next, the cells were washed 5 times with PBS. Firstly, cells were incubated for 1 h in medium containing 2.5 mM glucose (preincubation). Then, the medium was replaced by a medium containing either low glucose (2.5 mM glucose + tolbutamide) or high glucose (11.5 mM glucose + tolbutamide) for 1 h at 37 °C. The supernatants were collected to measure insulin secretion in response to the glucose. Tolbutamide partially depolarizes cells by decreasing their potassium permeability. This depolarization causes calcium outflow through the opening of voltage-dependent calcium channels and, as a result, insulin is released [21]. For this reason, tolbutamide was used to stimulate insulin release. Insulin levels were measured at 450 nm using an ultrasensitive rat/mouse insulin ELISA kit (Mercodia Inc, Sweden), and values were represented as insulin secretion (ng/mg protein) after normalizing to the total protein content (Pierce BCA, protein assay kit, Thermo Fisher Sci., USA).

### 2.6. Statistical analysis

The statistical analyses were performed using SPSS version 23.0 (SPSS, Chicago, IL, USA). Data were expressed as mean ± standard deviation. All experiments were repeated at least three times. Student’s t test was used and the results were considered to be significant at p < 0.05. All graphs were drawn using GraphPad Prism software version 8 (GraphPad Software, Inc., San Diego, CA, USA).

## 3. Results

### 3.1. LIF is required for pluripotency of mESCs

**Figure 1:**
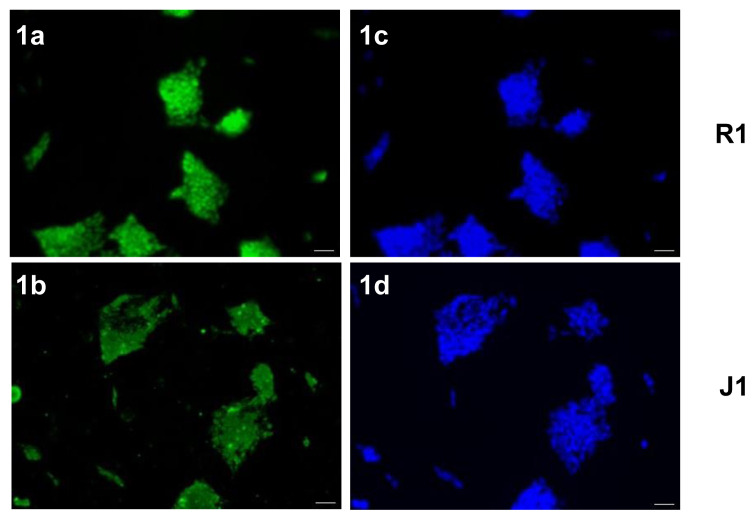
Sox-2 and Oct-4 proteins are expressed in the R1 mESCs. Immunocytochemical analysis of the pluripotency markers for (1a, 1c) Sox-2 and (b, d) Oct-4 in mESCs grown in the presence of LIF; (1c) and (1d) have the nuclei stained with DAPI. Scale bar: 50μm.

Pluripotent mESCs need the activity of different signaling pathways to maintain their capacity for self-renewal and differentiation into the 3 embryonic germ layers. The most important of these is the signaling pathway regulated via LIF/STAT3. We recently demonstrated LIF activity in our laboratory [15] with high expression levels of Oct-4, Nanog, Sox-2, and Klf4 genes, increased Stat3 phosphorylation, and strong ALP staining in mESCs cultured in the presence of LIF. In this study, we confirmed these results at the protein level using IF staining for pluripotency markers, Sox-2, and Oct-4. Mouse R1 ESCs that were cultured in the presence of LIF throughout the experiment as a control group were subjected to IF analysis using primary antibodies for Sox-2 and Oct-4 transcription factors followed by Alexa-Fluor 488-conjugated secondary and examined using a fluorescent microscope. The IF results together with DAPI staining revealed that mESCs expressed the pluripotency markers Sox-2 and Oct-4 in the presence of LIF in their nuclei (Figures 1a–1d).

### 3.2. In vitro differentiation of mESCs into IPCs is associated with morphological changes

**Figure 2:**
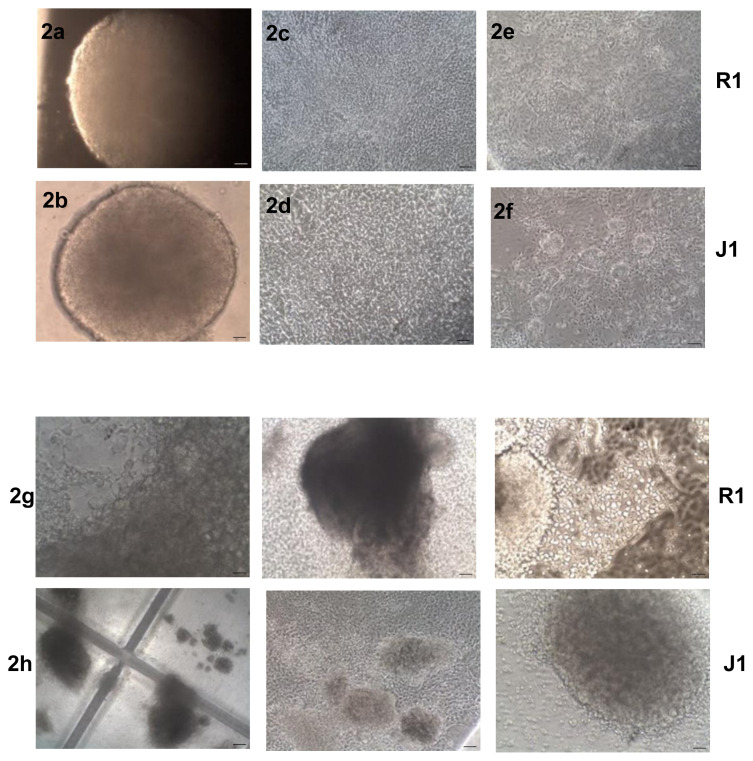
Microscopic visuals of the differentiation pathway of mESCs into IPCs on different culture days for the R1 and J1 cells. (2a, 2b) day 6; (2c, 2d) day 6 + 9; (2e, 2f) day 6 + 16; (2g, 2h) day 6 + 28. Scale bar: 50μm.

For the first stage of the differentiation procedure to induce EB formation, pluripotent mESCs were cultured using the hanging drops method for 6 days. The EBs exhibited a regular round-shaped morphology and smooth peripheral cell layer (Figures 2a and 2b). Then, the EBs were transferred onto gelatin-coated tissue-culture plates and cultured for 9 days for spontaneous differentiation (second stage). We observed epithelial-like and spindle-shaped cells around the EBs (Figures 2c and 2d). Next, the cells were transferred to Matrigel-coated plates and cultured in a pancreatic differentiation medium (third stage). At the stage of 6 + 16 days, the cells formed as loosely aggregated cell clusters (Figures 2e and 2f). Islet-like clusters were evident around the stage of 6 + 28 days (Figures 2g and 2h).

### 3.3. mESCs derived from IPCs express insulin

**Figure 3:**
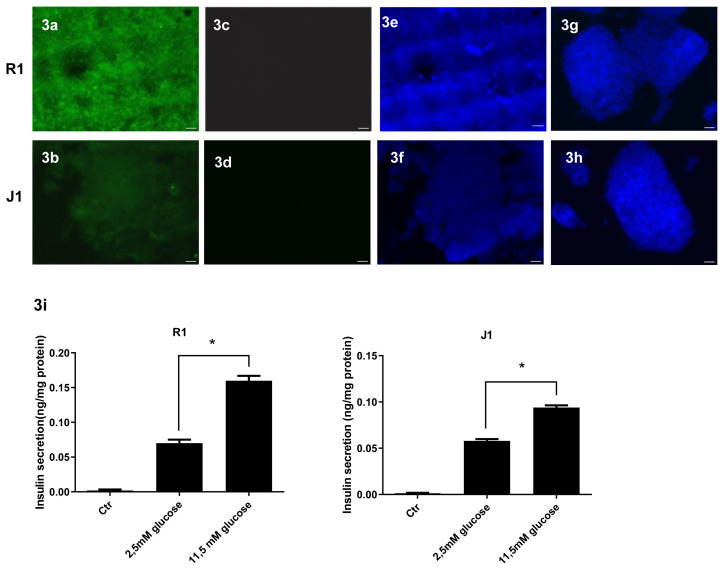
IF analysis and ELISA for the determination of IPCs. Insulin is detected in differentiated cells at stage 6 + 28 days (from R1 and J1 cells: 3a and 3b), whereas the mESCs exhibited no staining for insulin (R1 and J1 cells; 3c and 3d). Figures 3e–3h show nuclei labelled with DAPI. (3i) Glucose-stimulated insulin secretions in response to low (2.5 mM) and high (11.5 mM) glucose concentrations determined by ELISA at stage 6 + 28 days. Each value represents the mean ± SD. *p < 0.05, compared to 2.5 mM glucose concentration. Scale bar: 50μm.

Differentiation of mESCs into IPCs was successfully performed with the method described above. To confirm insulin production, IF staining was performed using a fluorescent labeled insulin antibody at the end of 6 + 28 days. The differentiated cells were observed to be insulin-positive (Figures 3a and 3b). The undifferentiated cells (mESCs) did not express insulin (Figures 3c and 3d). The nuclei were stained with DAPI (Figures 3e–3h).

### 3.4. mESCs derived from IPCs secrete insulin in response to glucose

To assess insulin secretion, we measured insulin release in response to a glucose challenge in IPCs on 6 + 28 days. The differentiated cells secreted insulin in response to both 2.5 mM and 11.5 mM glucose (v.s. mESCs). The differentiated cells incubated in 11.5 mM glucose secreted higher insulin levels than the cells incubated in 2.5 mM glucose (*p < 0.05) (Figure 3i).

### 3.5. Pancreas-specific genes are upregulated during differentiation of mESCs into IPCs

**Figure 4:**
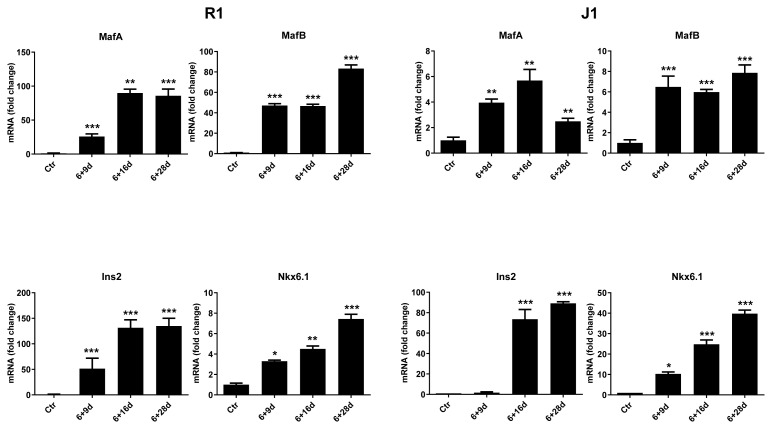
qPCR analyses of pancreas-specific genes. MafA, MafB, Ins2 and Nkx6.1 were upregulated in the differentiated cells at differentiation stages 6 + 9, 6 + 16, and 6 + 28 days. The Tubb5 gene was used as a housekeeping gene standard. *p < 0.05, **p < 0.01, ***p < 0.001 compared to the control undifferentiated R1 and J1 mESCs (Ctr).

To confirm the differentiation of mESCs into IPCs, the changes in the gene expression of some markers related to pancreatic development and maturation of β cells at certain stages of the differentiation process (6 + 9, 6 + 16, and 6 + 28 days) were examined by qPCR. mESCs cultured in the presence of LIF during the experiment were used as the control group. Significant increases were observed in MafA, MafB, Insulin (Ins2) and Nkx6.1 gene expressions in both cell lines (*p < 0.05) (**p < 0.01) (***p < 0.001) (Figure 4).

### 3.6. Pfkfb2 and Pfkfb3 expressions increased in the various stages of differentiation of mESCs into IPCs

**Figure 5:**
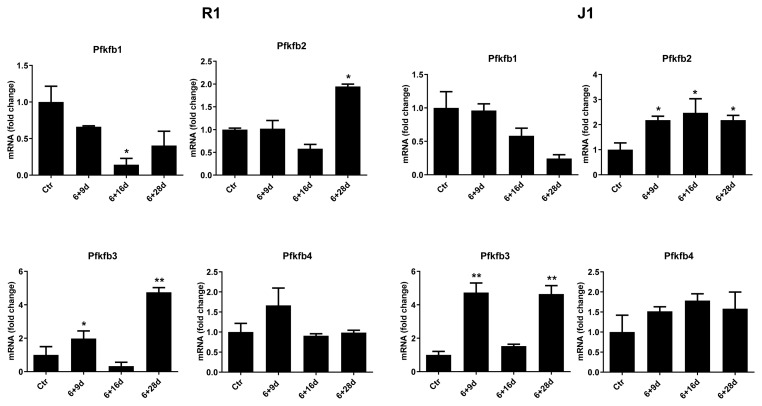
qPCR analyses of Pfkfb isoenzymes in differentiated cells. The Pfkfb2 gene was upregulated in various stages of differentiation from the R1 (6 + 28 days) and J1 (6 + 9, 6 + 16, and 6 + 28 days) cell lines (p < 0.05). Pfkfb3 expression increased in various stages of differentiation from the R1 (6 + 9 d (*p < 0.05) and 6 + 28 d (**p < 0.01)) and J1 (6 + 9 and 6 + 28 d (**p < 0.01)) cell lines. Undifferentiated R1 and J1 mESCs served as the control (Ctr). The Tubb5 gene was used as a housekeeping gene.

To examine the effect of differentiation on Pfkfb isoenzymes, qPCR was performed on lysates collected at stages 6 + 9, 6 + 16, and 6 + 28 days. mESCs grown in the presence of LIF throughout the experiment were used as the control group. Significant increases were observed especially in expressions of Pfkfb2 and Pfkfb3 isoforms in various stages of differentiation of the R1 and J1 cell lines (*p < 0.05) (**p < 0.01) (Figure 5). Tubb5 was used as the control gene.

## 4. Discussion

An essential role of the Pfkfb family of enzymes is to differentially regulate glycolysis and gluconeogenesis in the cytoplasm by PFK-1 [22]. There are 4 isoforms of these enzymes (Pfkfb1–4), which contain both kinase and phosphatase domains on the same polypeptide chain. The kinase activity of these enzymes catalyzes the formation of F2,6BP from F6P whereas the phosphatase activity catalyzes the conversion of F2,6BP to F6P. Among the 4 isoenzymes, Pfkfb3 exhibits the highest kinase/phosphatase ratio (740:1) and is the most studied isoform in tumor cells [12,16,17,23]. While glycolytic activity is known to increase in cells with a predominant total Pfkfb kinase activity, the reduced glycolytic rate is observed in cells with a high total Pfkfb phosphatase activity. Because of their crucial functions in glycolytic activity, researchers have largely focused on the roles of Pfkfb isoenzymes in tumor cell biology [24]. Studies on the roles of Pfkfb isoenzymes in mESCs are scarce. We recently reported that spontaneous differentiation of mESCs is associated with upregulation of Pfkfb3 expression, and that Pfkfb3 may be essential for the growth and differentiation of mESCs [15]. On the basis of our previous findings [15], we set out to study the changes in the expression of Pfkfb isoenzymes upon the differentiation of mESCs into IPCs in order to shed light on their potential roles during the different differentiation stages of mESCs.

There is currently no cure for T1DM, which is among the serious health problems in the world. In recent years, IPCs produced from stem cells have been a glimmer of hope for patients suffering from T1DM [5]. In order to mitigate problems associated with the use of stem cell-based therapies, it is of great importance to elucidate the cellular, genetic, and biochemical pathways that play critical roles in this differentiation. Studies have shown that IPCs are dependent on glycolytic activity for survival, and this dependence is associated with high levels of glycolytic enzymes [7,25]. These studies suggest that the Pfkfb isoenzymes, which play an important role in the control point of glycolysis, may also have roles in the metabolism and generation of IPCs from mESCs. Although the roles of Pfkfb isoenzymes in tumor cell metabolism have been well studied due to their relationship with glycolysis [12,26], studies focusing on their potential roles in metabolism and differentiation of mESCs are almost nonexistent [15]. In the current study, we found significant increases in the gene expression of Pfkfb2 and Pfkfb3 isoenzymes during the generation of IPCs from the R1 and J1 mESCs. Pfkfb3 has been shown to be highly expressed in various tumor cells, including pancreatic, breast, and gastric cancer [26–28]. A recent study from our research team [29] showed the need for the Pfkfb2 isoenzyme in the proliferation and glycolysis of pancreatic cancer cells. Moreover, Pfkfb3 expression increases by 1.73-fold in the pancreatic β cells of mice that were made diabetic by streptozotocin [30]. In our recent study [15], we reported that the expression of the Pfkfb3 isoenzyme was markedly increased in spontaneously differentiated mESCs, and this elevation is required for the growth of early differentiated mESCs. Chesney et al. [11] reported that the genomic deletion of the Pfkfb3 gene results in embryonic lethality in mice. Pegoraro et al. [31] demonstrated that the tissue-specific, dynamic and complementary expression patterns of Pfkfb genes play an essential role in the developmental stage of *Xenopus laevis* (African clawed frog) embryos from blastula to tadpole stages. In another study by the same team [32], Pfkfb4 has been shown to play an important role in frog embryonic development and the differentiation of progenitor cells. There are also several studies suggesting the participation of Pfkfb3 in the differentiation of epidermal keratinocytes [14] and preadipocytes [33]. However, our study constitutes a first attempt to examine the changes in Pfkfb isoenzymes during the various stages of differentiation of mESCs into IPCs. Swisa et al. [8] demonstrated that inhibition of the LKB1 enzyme, which is the activator of AMPK stimulating by AMP, an important glycolysis activator, causes an increase in glucose-stimulated insulin secretion from pancreatic β cells. Previous studies have also reported that some transcription factors and proteins related to pancreatic stem cells may have important roles in the generation of IPCs from mESCs. The transcription factors Nkx6.1 and Pdx1 have been shown to be crucial in the differentiation of IPCs from progenitor cells [2]. Recent reports have also showed that transcription factors such as Glut2, Ins, C-peptide, MafA, and MafB are also important for pancreatic differentiation of ESCs [25]. In the current study, the increases in Pfkfb2 and pfkfb3 gene expression at the various stages of the differentiation of mESCs into IPCs are promising in the sense that these isoenzymes may also have roles in this process. The observation that both Pfkfb2 and Pfkfb3 expressions changed similarly in differentiating mESCs suggests that these 2 isoenzymes may support each other’s function during differentiation. However, we cannot rule out the possibility that these isoenzymes may have nonreduntant functions that regulate different aspects of mESC differentiation. Therefore, future studies should focus on delineating the requirement of each isoenzyme in the differentiation of mESCs. A key limitation of this study is that differentiation of mESCs into IPCs is associated with potential changes in the subcellular localizations of Pfkfb isoenyzmes, which is known to affect the activity of these enzymes [16].

In light of our findings, studies on the potential roles of Pfkfb2 and Pfkfb3 isoenzymes in the differentiation of mESCs into IPCs using various tools such as CRISPR/Cas9-mediated gene inactivation are warranted, which may contribute to a better understanding of DM and lead to the development novel treatment strategies. The present study provides the first evidence that the Pfkfb2 and Pfkfb3 isoenzymes may have roles in the differentiation of mESCs into IPCs. However, further studies are needed to fully clarify whether Pfkfb2 alone, Pfkfb3 alone, or an additive effect of both isoenzymes play a more potent role in the differentiation of mESCs into IPCs.
